# 超高效液相色谱-三重四极杆质谱法测定干茶中35种吡咯里西啶生物碱

**DOI:** 10.3724/SP.J.1123.2025.01020

**Published:** 2025-12-08

**Authors:** Lu DONG, Xiaolin ZHANG, Chen YANG, Jingyan ZHAO, Xuemei WANG, Yingqian CHU, Xuehua LI

**Affiliations:** 1.大连理工大学环境学院，工业生态与环境工程重点实验室，辽宁 大连 116024; 1. Key Laboratory of Industrial Ecology and Environmental Engineering，School of Environment，Dalian University of Technology，Dalian 116024，China; 2.大连海关技术中心，辽宁 大连 116001; 2. Technology Center of Dalian Customs District，Dalian 116001，China

**Keywords:** 吡咯里西啶生物碱, 超高效液相色谱-三重四极杆质谱法, 茶叶, pyrrolizidine alkaloids （PAs）, ultra-high performance liquid chromatography-triple quadrupole mass spectrometry （UHPLC-MS/MS）, tea

## Abstract

吡咯里西啶生物碱（PAs）是一类植物内源产生的次生代谢产物，具有肝毒性、遗传毒性和致癌性等毒性作用。现有研究覆盖PAs种类有限，且由于同分异构体色谱分离难，大多研究采用加和定量策略。因此，本研究基于超高效液相色谱-三重四极杆质谱法（UHPLC-MS/MS）建立了干茶样本中欧盟法规限量的35种PAs的分析方法，其中有33种目标化合物获得了色谱分离，可以实现单独定量，有2种异构体共流出，进行加和定量。在电喷雾离子源（ESI）正离子模式下电离、多反应监测（MRM）模式下扫描，35种PAs在各自范围内呈现良好的线性关系（*r* ^2^>0.99），方法的检出限（LOD）和定量限（LOQ）分别为0.2~8.0 µg/kg和0.5~25.0 µg/kg。在1、2和5倍LOQ加标水平下，茶叶基质中均有89%以上的目标化合物平均回收率为70%~130%，3次平行实验相对标准偏差（RSD）小于20%（蓝蓟定和毛果天芥菜碱RSD小于30%）。应用该方法对21份黑茶和30份红茶样本进行检测，黑茶样本中35种PAs检出率为19.05%，总含量为5.07~15.48 µg/kg，红茶样本中无PAs检出。所建立的分析方法可为茶叶样本中PAs的组成、含量及风险评估提供技术支撑。

## Abstract (en)

Pyrrolizidine alkaloids （PAs） are natural toxins widely distributed in plants， which naturally occur in about 3% of the world’s flowering plants. To date， more than 660 PAs and their nitrogen oxides have been identified in over 6 000 plants. Some PAs are hepatotoxic， genotoxic and tumorigenic， posing significant health risks to humans. These alkaloids are commonly detected as contaminants in various food products， including tea， grains， milk， honey， as well as plant-derived pharmaceuticals and dietary supplements. Currently， most studies on the quantitative methods for PAs focus on a limited number of PAs and employ an additive quantification strategy， largely due to the challenges associated with chromatographic separation of isomers. These approaches limit the ability to assess exposure and health risk accurately. Herein， a method was established to quantify 35 PAs individually in dried tea samples using ultra-high performance liquid chromatography-triple quadrupole mass spectrometry （UHPLC-MS/MS） . The 35 target compounds were divided into two groups. The first group included 30 PAs， while the second group consisted of 5 PAs. These compounds were separated on Waters Acquity BEH C18 （150 mm×2.1 mm， 1.7 μm） and Thermo Acclaim^TM^ C30 （150 mm×2.1 mm， 3.0 μm） chromatographic columns， respectively. Mobile phases were H_2_O containning 5 mmol/L ammonium formate and 0.13% formic acid （pH=3） and methanol-acetonitrile （4∶6， volume raio） containing 0.1% formic acid. The 33 target compounds were separated and 2 isomers co-eluted. Under positive-ion electrospray ionization （ESI） and multiple reaction monitoring （MRM） mode， target compounds were quantified using the external standard method with the matrix-matched standard curve. The results demonstrated that all target compounds showed good linearity （*r* ^2^>0.99） in their respective mass concentration ranges. The limits of detection （LODs） and quantification （LOQs） of the method were in the range of 0.2-8.0 µg/kg and 0.5-25.0 µg/kg， respectively. The average recoveries of more than 89% compounds were in the range of 70%-130% at spiked levels of 1， 2， and 5 fold-LOQs and the relative standard deviations （RSDs） were less than 20% （RSDs of lasiocarpine and echimidine were less than 30%）. Moreover， the quantitative method of the 35 PAs was applied to 21 dark tea and 30 black tea samples from Yunnan and Fujian Province， where PAs were detected in 4 dark samples with total contents ranging from 5.07 to 15.48 µg/kg and were not detected in black tea samples. The detection rate of PAs in dark tea samples was higher than that of black tea samples. That might be because most dark tea was processed from fresh leaves picked by machines， and this attribution could be associated with the mixing of weeds containing PAs during tea harvesting. And the detected concentrations of all samples were lower than the maximum levels of tea in European Union regulations， indicating that the tea samples involved in this research are basically safe. In brief， the quantitative method of the 35 PAs facilitates the analysis of the occurrence， composition and potential risks in tea samples.

吡咯里西啶生物碱（pyrrolizidine alkaloids， PAs）是一类植物内源产生的次生代谢产物。其存在非常广泛，全世界约有6 000种植物可以产生PAs，占所有开花植物的3%，目前有660余种PAs（包含吡咯里西啶生物碱氮氧化物PANOs）已被识别^［1-3］^。PAs在植物的化学防御系统中起着关键作用，帮助抵御食草动物、昆虫及病原体的侵害^［4］^。PAs是由千里光次碱和千里光次酸两个基本部分组成的酯类物质。根据千里光次碱的吡咯环结构中1，2位碳原子之间形成的键是否饱和，PAs可分为饱和型PAs和不饱和型PAs。饱和型PAs通常无毒或毒性较弱，而不饱和型PAs由于其不可忽视的毒性成为关注的热点。根据千里光次碱结构的差异，不饱和型PAs又可进一步分为倒千里光碱型（retronecine-type PAs）和奥索千里光裂碱型（otonecine-type PAs） （图1）^［5］^。PAs具有多种毒性作用，包括肝毒性、遗传毒性和致癌性等^［6-9］^，但不同PAs间毒性差异很大。Ruan等^［5］^把发挥毒性作用的中间体吡咯-蛋白加合物作为生物标志物来评估PAs的毒性程度，结果证明倒千里光碱型PAs的毒性大于奥索千里光裂碱型。同时进一步对不同结构的倒千里光碱型PAs进行对比发现，开环二酯的代谢活化率和毒性远高于大环二酯。因此，发展多种PAs单独定量的分析技术是提升PAs健康风险评估可靠性和准确性的有效手段。

**图1:**
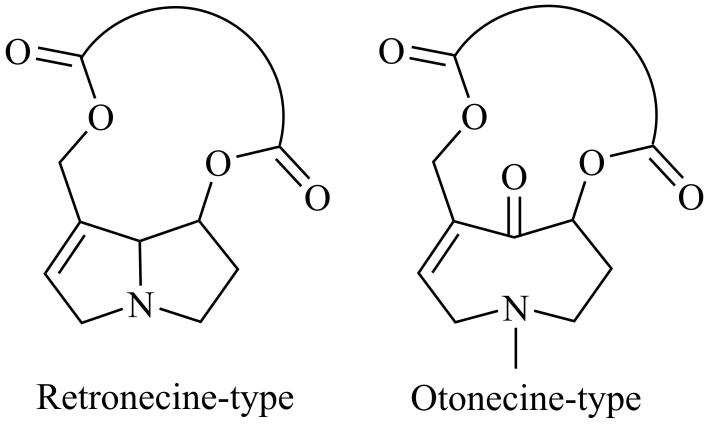
倒千里光碱型和奥索千里光裂碱型PAs的化学结构

由于PAs暴露对人体健康存在不良影响，欧盟颁布了《欧盟委员会条例（欧盟）》（（EU）2023/915）^［10］^，规定了干茶样本中35种PAs总和的最高含量为150 μg/kg。但当前PAs检测方法研究普遍存在目标物覆盖不足的问题，Jiao等^［11，12］^分析了我国安徽省茶叶和茶园杂草中15种PAs的存在水平，目标物质仅涵盖欧盟法规限量中的13种；陈言凯等^［13］^虽开发了同时测定乳粉及液体乳中26种PAs的分析方法，但法规物质覆盖率仅54.3% （19/35）；Rizzo等^［14］^使用高分辨质谱建立了118种PAs的筛查方法，但仅能对28种PAs进行准确定量；张新娜等^［15］^将甘草中检测目标扩展至34种PAs，但其中也仅包含了法规严格限量的24种，且未考察全缘千里光碱等共洗脱干扰物。姚蕾珺等^［16］^和黄琼^［17］^分别建立了茶叶中27种PAs和牛奶中30种PAs的定量分析方法，但目标物均仅涵盖了法规中规定的21种PAs，且忽略了多个可能与目标PAs共流出的物质，如可能与石松胺和促黑激素共流出的凌德草碱、刺凌德草碱和大尾摇碱等，这对目标PAs的定量存在干扰。此外，PAs中含有多种同分异构体，色谱分离较为困难。因此对于难以色谱分离的同分异构体，大多数研究只能以两种或更多种化合物的加和浓度进行报道。万静宜等^［18］^分析了我国市售茶叶样本中21种PAs的存在水平，其中19种实现了色谱分离；Jansons等^［19］^基于纳流液相色谱-串联质谱法建立了30种PAs的分析方法，其中仅24种实现了完全色谱分离和单独定量；Peloso等^［20］^对意大利市场上600多种食品中的16种目标PAs进行了单独定量，并对其余19种难以色谱分离的PAs进行分组加和定量。但值得注意的是，对多种目标物质进行加和定量可能存在交叉干扰的问题，不利于后续的健康风险评估及科学管控。

茶是一种消费最广泛的非酒精饮料，茶叶样本中频繁检出PAs，欧盟食品和饲料快速预警系统中经常发布有关茶叶及其制品中检出高含量PAs的报道^［21-23］^，因此饮茶可能导致人群暴露于PAs进而产生潜在健康风险^［12，24］^。但我国作为茶叶生产和消费大国，茶叶中PAs存在水平的相关研究十分有限，分析方法也有待完善^［25，26］^。因此，本研究基于超高效液相色谱-三重四极杆质谱法（ultra-high performance liquid chromatography-triple quadrupole mass spectrometry， UHPLC-MS/MS）开发了欧盟法规限量中35种PAs的定量分析方法，并采用干茶样本进行了验证。将35种目标PAs分为两组分别测定，最终有33种目标化合物获得了色谱分离，可以单独定量，有2种化合物（即促黑激素氮氧化物和大尾摇碱氮氧化物）共流出，进行加和定量。应用本研究开发的方法分析了我国云南和福建省21份黑茶和30份红茶样本中PAs的存在水平，在4份样本中检出了PAs。本研究建立的分析方法可为茶叶中PAs的组成、含量及风险评估提供技术支撑。

## 1 实验部分

### 1.1 仪器、试剂与材料

LC-30 AD液相色谱系统（日本Shimadzu公司）、QTRAP 6500质谱仪（美国AB SCIEX公司）；Quintix 612精度为0.01 g分析天平（德国Sartorius公司）；KQ-500DE超声萃取仪（昆山超声仪器有限公司）；3-18K高速冷冻离心机（美国Sigma公司）；SPE-12固相萃取装置（美国Supelco公司）；N-EVAP112氮吹浓缩仪（美国Organomation公司）；混合型阳离子交换固相萃取小柱（Oasis MCX小柱， 60 mg， 3 mL， 美国Waters公司）；Arium超纯水机（德国Sartorius公司）；0.22 μm滤膜（上海安谱实验科技股份有限公司）。

甲醇、乙腈、甲酸铵、甲酸、硫酸、氨水均购于德国Merck公司；纯净水购于广州屈臣氏食品饮料有限公司；21份黑茶样本取自云南省4个茶叶产区（勐海茶区、易武茶区、普洱茶区、临沧茶区）种植园；30份红茶样本分别取自云南省（18份）和福建省（12份）茶叶种植园；35种PAs标准品（见表1）购于天津阿尔塔科技股份有限公司，质量浓度均为100 μg/mL（乙腈溶液中）。

**表1:** 35种目标PAs的信息

No.	Compound	Abbr.	Chinese name	CAS No.	Molecular formula
1	intermedine	Im	促黑激素	10285-06-0	C_15_H_25_NO_5_
2	rinderine	Rn	凌德草碱	6029-84-1	C_15_H_25_NO_5_
3	indicine	Id	大尾摇碱	480-82-0	C_15_H_25_NO_5_
4	lycopsamine	La	石松胺	10285-07-1	C_15_H_25_NO_5_
5	echinatine	En	刺凌德草碱	480-83-1	C_15_H_25_NO_5_
6	heliotrine	Hn	天芥菜碱	303-33-3	C_16_H_27_NO_5_
7	rinderine-*N*-oxide	RnN	凌德草碱氮氧化物	137821-16-0	C_15_H_25_NO_6_
8	echinatine-*N*-oxide	EnN	刺凌德草碱氮氧化物	20267-93-0	C_15_H_25_NO_6_
9	intermedine-*N*-oxide	ImN	促黑激素氮氧化物	95462-14-9	C_15_H_25_NO_6_
10	indicine-*N*-oxide	IdN	大尾摇碱氮氧化物	41708-76-3	C_15_H_25_NO_6_
11	lycopsamine-*N*-oxide	LaN	石松碱胺氮氧化物	95462-15-0	C_15_H_25_NO_6_
12	europine	Eu	欧天芥菜碱	570-19-4	C_16_H_27_NO_6_
13	heliotrine-*N*-oxide	HnN	天芥菜碱氮氧化物	6209-65-0	C_16_H_27_NO_6_
14	seneciphylline	Sp	千里光菲林	480-81-9	C_18_H_23_NO_5_
15	spartioidine	St	鹰爪千里光碱	520-59-2	C_18_H_23_NO_5_
16	integerrimine	Ir	全缘千里光碱	480-79-5	C_18_H_25_NO_5_
17	senecivernine	Sv	春千里光碱	72755-25-0	C_18_H_25_NO_5_
18	senecionine	Sc	千里光宁碱	130-01-8	C_18_H_25_NO_5_
19	europine-*N*-oxide	EuN	欧天芥菜碱氮氧化物	65582-53-8	C_16_H_27_NO_7_
20	spartioidine-*N*-oxide	StN	鹰爪千里光碱氮氧化物	171038165（CID）	C_18_H_23_NO_6_
21	seneciphylline-*N*-oxide	SpN	千里光菲林氮氧化物	38710-26-8	C_18_H_23_NO_6_
22	senecivernine-*N*-oxide	SvN	春千里光碱氮氧化物	101687-28-9	C_18_H_25_NO_6_
23	integerrimine-*N*-oxide	IrN	全缘千里光碱氮氧化物	85955-28-8	C_18_H_25_NO_6_
24	senecionine-*N*-oxide	ScN	千里光宁碱氮氧化物	13268-67-2	C_18_H_25_NO_6_
25	usaramine	Us	光萼野百合碱	15503-87-4	C_18_H_25_NO_6_
26	retrorsine	Re	倒千里光碱	480-54-6	C_18_H_25_NO^6^
27	senkirkine	Sk	克氏千里光碱	2318-18-5	C_19_H_27_NO_6_
28	retrorsine-*N*-oxide	ReN	倒千里光碱氮氧化物	15503-86-3	C_18_H_25_NO_7_
29	usaramine-*N*-oxide	UsN	光萼野百合碱氮氧化物	117020-54-9	C_18_H_25_NO_7_
30	heliosupine	Hs	天芥菜平	32728-78-2	C_20_H_31_NO_7_
31	echimidine	Em	蓝蓟定	520-68-3	C_20_H_31_NO_7_
32	lasiocarpine	Lc	毛果天芥菜碱	303-34-4	C_21_H_33_NO_7_
33	echimidine-*N*-oxide	EmN	蓝蓟定氮氧化物	41093-89-4	C_20_H_31_NO_8_
34	heliosupine-*N*-oxide	HsN	天芥菜平氮氧化物	31701-88-9	C_20_H_31_NO_8_
35	lasiocarpine-*N*-oxide	LcN	毛果天芥菜碱氮氧化物	127-30-0	C_21_H_33_NO_8_

### 1.2 标准溶液配制

分别准确吸取20 μL 35种单标储备液于10 mL容量瓶中，后用乙腈定容至10 mL以配制200 ng/mL混合标准储备液。使用乙腈将上述混合标准储备液逐级稀释成0.1、0.2、0.5、1、2、5、10、20、50 ng/mL的一系列混合标准溶液，于-18 ℃冰箱中避光保存。

取500 μL处理后的干茶样品溶液，加入适量200 ng/mL 混合标准储备液配制成一系列的PAs混合基质匹配标准溶液，质量浓度分别为0.1、0.2、0.5、1、2、5、10、20、50 ng/mL，于-18 ℃冰箱中避光保存。

### 1.3 样品前处理

前处理方法在前人已发表研究^［18，27-29］^的基础上进行了改进，具体步骤如下：准确称量（2.00±0.01） g干茶粉末样品置于离心管中，加入20 mL含0.05 mol/L硫酸的甲醇-水（1∶1，体积比）提取液，涡旋30 s后超声萃取10 min，将提取物离心（10 000 r/min， 5 min）后取1 mL上清液加载到已用1.5 mL甲醇和1.5 mL水活化的Waters Oasis MCX（60 mg， 3 mL）固相萃取柱上，接着用2 mL水和2 mL甲醇淋洗SPE小柱，用2 mL 含2.5%氨水的甲醇溶液洗脱PAs。将洗脱的溶液置于氮气下干燥，后用500 μL初始流动相溶液复溶，过0.22 μm滤膜后上机测定。

### 1.4 UHPLC-MS/MS检测条件

本研究将35种目标PAs分为两组分别测定。第一组包括30种目标化合物，第二组包括其余5种同分异构体目标化合物（见表2）。第一组化合物用方法1分析，第二组化合物用方法2分析。

**表2:** 35种PAs的质谱参数

No.	Compound	Retention time/min	Parent ion （*m*/*z*）	Product ions （*m*/*z*）	DP/V	CEs/eV
**Method 1**						
1	Hn	15.85	314.2	138.1^*^， 156.1	90	27， 33
2	RnN	9.64	316.2	111.1^*^， 172.1	125	54， 35
3	EnN	9.90	316.2	111.1^*^， 172.1	120	47， 36
4	ImN	10.50	316.2	138.1^*^， 172.1	100	38， 38
5	IdN	10.50	316.2	172.1^*^， 94.2	105	36， 61
6	LaN	10.99	316.2	138.1^*^， 172.1	115	40， 38
7	Eu	8.14	330.0	138.1^*^， 254.1	100	29， 24
8	HnN	17.53	330.0	111.1^*^， 172.1	140	48， 37
9	St	15.99	334.2	120.3^*^， 138.3	110	36， 37
10	Sp	16.49	334.2	120.3^*^， 306.1	140	36， 37
11	Ir	20.96	336.1	94.2^*^， 120.3	135	44， 38
12	Sv	21.27	336.1	120.3^*^， 138.1	140	39， 41
13	Sc	21.78	336.1	120.3^*^， 138.1	100	38， 39
14	EuN	9.19	346.0	172.1^*^， 328.3	110	38， 30
15	StN	17.46	350.2	118.2^*^， 94.2	140	38， 72
16	SpN	18.11	350.2	120.3^*^， 118.2	150	40， 39
17	Us	14.09	352.3	94.2^*^， 120.3	130	36， 41
18	Re	14.56	352.3	120.3^*^， 324.1	140	41， 36
19	SvN	21.90	352.3	118.2^*^， 120.3	150	40， 43
20	IrN	22.15	352.3	120.3^*^， 94.2	150	44， 66
21	ScN	22.76	352.3	118.2^*^， 120.3	150	41， 41
22	Sk	25.07	366.2	150.1^*^， 168.1	120	35， 46
23	ReN	14.93	368.2	118.2^*^， 136.2	165	39， 45
24	UsN	15.12	368.2	120.3^*^， 94.2	170	40， 73
25	Hs	25.00	398.2	120.3^*^， 220.2	100	35， 25
26	Em	25.24	398.2	120.3^*^， 220.2	100	33， 23
27	Lc	28.94	412.3	120.3^*^， 336.3	115	34， 23
28	EmN	24.85	414.2	254.1^*^， 352.2	120	39， 32
29	HsN	26.86	414.2	94.2^*^， 254.1	140	70， 39
30	LcN	30.60	428.2	136.2^*^， 254.1	130	42， 37
**Method 2**						
31	Im	9.73	300.2	138.1^*^， 156.1	67	26， 36
32	Rn	10.54	300.2	138.1^*^， 156.1	105	29， 37
33	Id	11.02	300.2	138.1^*^， 94.2	110	33， 33
34	La	11.44	300.2	156.1^*^， 138.1	70	37， 27
35	En	12.13	300.2	138.1^*^， 156.1	90	29， 35

DP： declustering potential； CE： collision energy； * quantitative ion.

#### 1.4.1 方法1

色谱柱：Waters Acquity BEH C18（150 mm×2.1 mm，1.7 μm，美国Waters公司）；流动相A：含5 mmol/L甲酸铵的0.13%甲酸水溶液（pH=3），流动相B：含0.1%甲酸的甲醇-乙腈（4∶6，体积比）溶液；梯度洗脱程序：0~0.01 min，5%B；0.01~18 min，5%B~15%B；18~30 min，15%B~35%B；30~31 min，35%B~95%B；31~33 min，95%B；33~34 min，95%B~5%B；34~35 min，5%B。流速为0.3 mL/min；进样量为5 μL；柱温为40 ℃。

离子源：电喷雾电离（ESI）源，正离子模式；扫描方式：多反应监测模式（MRM）；气帘气（curtain gas）流速为30 L/min，喷雾电压（ionspray voltage）为5 500 V，离子源温度为550 ℃。目标化合物的保留时间、母离子、子离子、去簇电压（declustering potential， DP）、碰撞能量（collision energy， CE）等质谱信息见表2。使用UHPLC-MS/MS配套的Analyst软件进行数据采集，MultiQuant软件进行数据分析。

#### 1.4.2 方法2

色谱柱：Thermo Acclaim^TM^ C30（150 mm×2.1 mm，3.0 μm，美国Thermo公司）；流动相A：含5 mmol/L甲酸铵的0.13%甲酸水溶液（pH=3），流动相B：含0.1%甲酸的甲醇-乙腈（4∶6，体积比）溶液；梯度洗脱程序：0~1.0 min，1%B；1.0~16.0 min，1%B~4%B；16.0~16.1 min，4%B~95%B；16.1~19.5 min，95%B；19.5~19.6 min，95%B~1%B。流速为0.4 mL/min；进样量为5 μL；柱温为40 ℃。

质谱条件同1.4.1节。

## 2 结果与讨论

### 2.1 方法优化

#### 2.1.1 色谱条件的优化

35种目标PAs中有10组同分异构体物质，其中分子式为C_15_H_25_NO_5_（Im、Rn、Id、La、En）、C_15_H_25_NO_6_（RnN、EnN、ImN、IdN、LaN）和C_18_H_25_NO_6_（SvN、IrN、ScN、Us、Re）各有5种同分异构体，分子式为C_18_H_25_NO_5_（Ir、Sv、Sc）有3种同分异构体，分子式为C_16_H_27_NO_6_（Eu、HnN）、C_18_H_23_NO_5_（Sp、St）、C_18_H_23_NO_6_（SpN、StN）、C_18_H_25_NO_7_（ReN、UsN）、C_20_H_31_NO_7_（Hs、Em）和C_20_H_31_NO_8_（EmN、HsN）各有2种同分异构体。同分异构体质谱碎片一致，无法在质谱的MRM模式下进行分离，因此需要优化色谱条件将同分异构体完全分离后才能进行准确定性和定量。色谱柱是色谱分离的重要影响因素，本研究分别考察了色谱柱Waters Acquity BEH C18（150 mm×2.1 mm，1.7 μm）、Waters CORTECS^TM^ UPLC C18+（150 mm×2.1 mm，1.6 μm）、Waters Acquity CSH^TM^ C18（100 mm×2.1 mm，1.7 μm）、Waters Acquity UPLC HSS T3（100 mm×2.1 mm，1.8 μm）、Waters Acquity HSS PFP（100 mm×2.1 mm，1.8 μm）、Thermo Hypersil gold C18 （100 mm×2.1 mm，1.9 μm）、Agilent XDB C18（100 mm×2.1 mm，1.8 μm）和Agilent Poroshell EC-C18（150 mm×2.1 mm，2.7 μm），分别标记为柱1~8，对应的35种PAs的分离情况如图2所示。可以看出，柱1~6均对目标物具有较好的保留（图2a~2f），而柱7和柱8无分离优势（图2g和2h）。进而通过对多组同分异构体的提取离子流色谱图分析发现，Waters Acquity BEH C18色谱柱（150 mm×2.1 mm，1.7 μm）对分子式为C_18_H_25_NO_5_的3个同分异构体和分子式为C_18_H_25_NO_7_的2个同分异构体有显著的分离优势，因此选择柱1作为本研究的色谱柱。但分子式为C_15_H_25_NO_5_的5个同分异构体在已有8根色谱柱上均不能实现完全的色谱分离。针对该组同分异构体，本研究发现具有更长碳链填料和更大粒径的Thermo Acclaim^TM^色谱柱 C30（150 mm×2.1 mm，3.0 μm）表现出明显的分离优势，可以实现对5个化合物完全的基线分离。因此使用该色谱柱对Im、Rn、Id、La和En进行色谱分离。

**图2:**
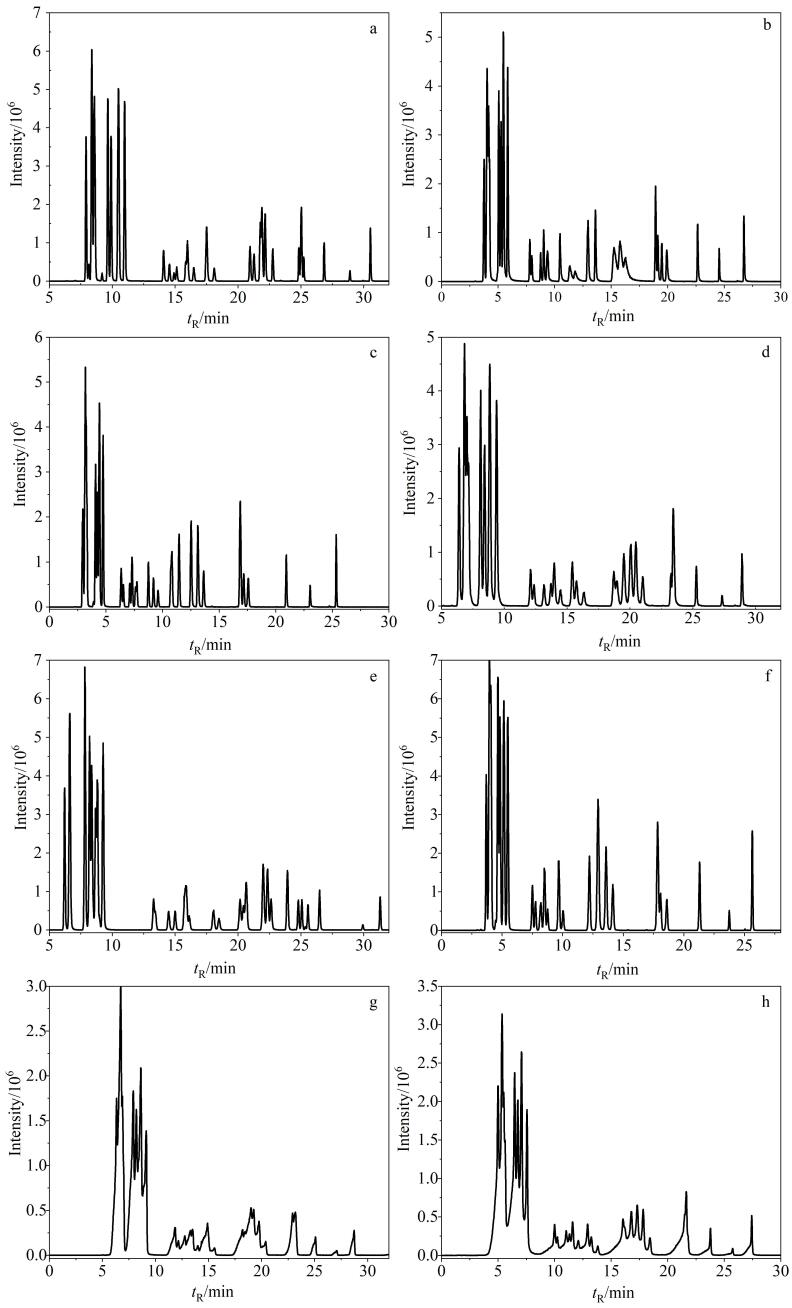
采用不同色谱柱时35种PAs的总离子流色谱图

**图3:**
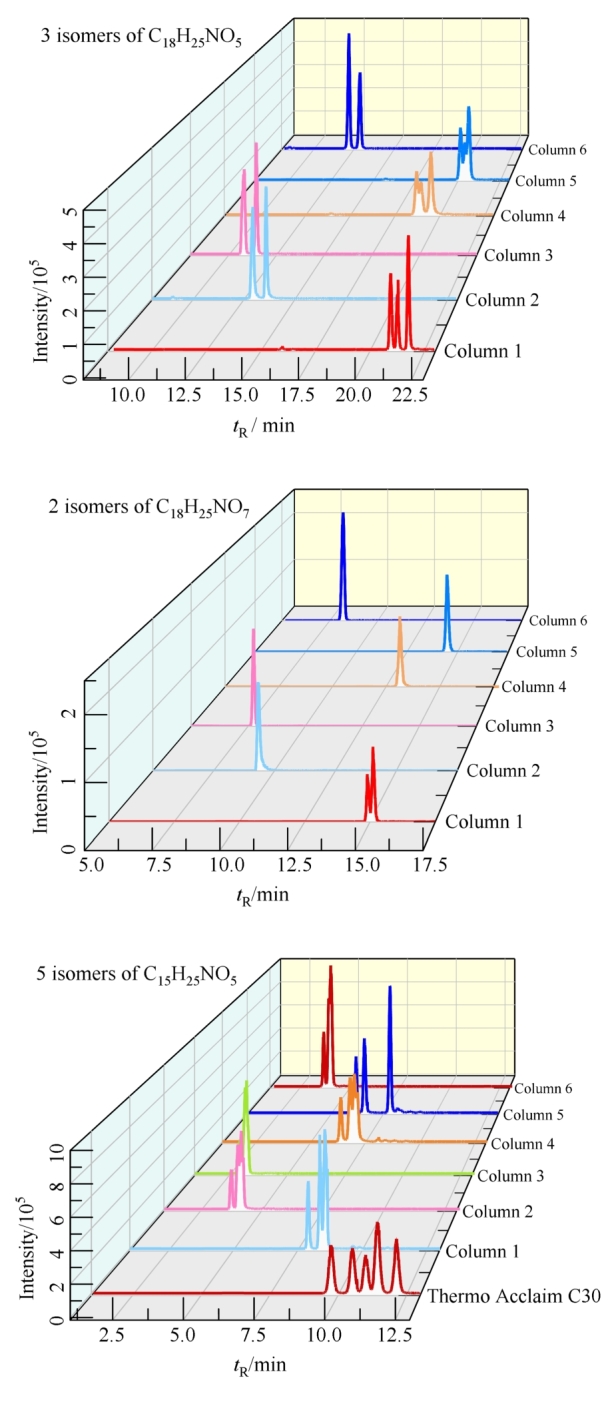
采用不同色谱柱时3组同分异构体的提取离子流色谱图

为了提高色谱分离效果和目标化合物的检测灵敏度，并获得良好的峰形，实验对流动相体系进行了优化。分别考察了甲醇、乙腈和不同比例的甲醇-乙腈作为流动相有机相时的色谱分离效果。结果表明，甲醇-水作为流动相时，PAs出峰时间较晚且对C_18_H_25_NO_7_（ReN、UsN）同分异构体分离效果不理想，出现多个肩峰和无法达到完全基线分离的情况。而在乙腈-水流动相体系下，30 min内可以实现所有PAs的有效分离，但C_18_H_25_NO_6_（SvN、IrN、ScN、Us、Re）同分异构体的分离度较差，噪声大，灵敏度低。因此实验对比了不同比例甲醇-乙腈对色谱分离的影响。结果显示，有机相为甲醇-乙腈（4∶6，体积比）时，目标化合物色谱分离效果最好。此外，为改善色谱峰形，在流动相的水相中添加5 mmol/L甲酸铵缓冲盐，色谱峰形在一定程度上得到改善，拖尾减少。在水相和有机相中均添加甲酸后，目标化合物离子化效率和相应强度提高。综合考虑，选择流动相A为含5 mmol/L甲酸铵的0.13%甲酸水溶液（pH=3），流动相B为含0.1%甲酸的甲醇-乙腈（4∶6，体积比）的流动相体系。在该流动相体系下35种PAs具有较好的响应和峰形，且通过优化梯度洗脱程序，能通过两种方法实现欧盟法规限量中35种PAs最大程度的基线分离。

#### 2.1.2 质谱条件的优化

采用单标单针进样的方式，将35种20 ng/mL的PAs标准储备液分别直接注入质谱仪中用于质谱条件的优化。在ESI源正离子模式下进行扫描（*m/z* 100~500）选择母离子，确定35种目标物的母离子峰均为［M+H］^+^。在确定母离子的基础上进行子离子扫描，获得各化合物的碎片离子，通过比较响应值选择2个子离子碎片，以响应值高的通道作为定量离子通道，响应值略低的作为定性离子通道。利用母离子的信号强度优化DP，子离子的信号强度优化CE。在最佳质谱条件的基础上建立了MRM扫描模式进行信息采集。目标PAs的保留时间和质谱参数信息如表2所示。

在最优的色谱和质谱条件下得到35种PAs的提取离子流色谱图见图4。

**图4:**
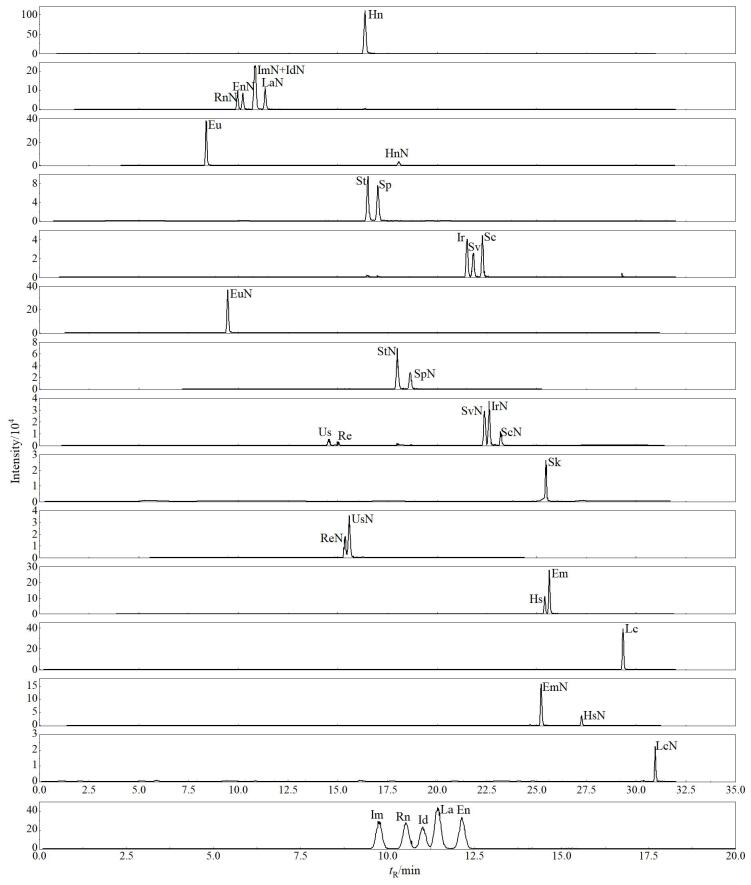
35种PAs的提取离子流色谱图

### 2.2 基质效应

与溶剂相比，茶叶基质复杂，含有多种干扰PAs测定的其他物质，这种干扰称为基质效应（ME）。采用基质匹配标准曲线与溶剂标准曲线斜率的比值计算基质效应。ME值大于100%表明存在基质增强效应；ME值小于100%表明存在基质抑制效应；ME值为80%~120%时，表明基质效应影响较小。本研究以茶叶样本为例，35种PAs的ME范围为16.32%~62.82%（如表3所示）。表明所有化合物均存在较强的基质抑制效应，因此采用基质匹配标准曲线对目标化合物进行定量分析。

**表3:** 35种PAs的线性范围、回归方程、相关系数（*r* ^2^）、检出限、定量限和基质效应

No.	Compound	Linear range/（ng/mL）	Linear equation	*r* ^2^	LOD/（μg/kg）	LOQ/（μg/kg）	ME/%
1	Im	1.0‒50	*y*=64396.8*x*‒4097.0	0.99957	1.5	5.0	58.78
2	Rn	1.0‒50	*y*=54473.0*x*+4620.6	0.99975	1.5	5.0	60.23
3	Id	1.0‒50	*y*=93011.4*x*+11787.3	0.99997	1.5	5.0	59.29
4	La	1.0‒50	*y*=141403.0*x*+16000.25	0.99989	1.5	5.0	62.63
5	En	1.0‒50	*y*=102298.0*x*+14059.8	0.99943	1.5	5.0	62.82
6	Hn	0.5‒50	*y*=113915.0*x*+771.1	0.99959	0.8	2.5	30.17
7	RnN	0.5‒50	*y*=17860.4*x*+5398.1	0.99882	0.8	2.5	48.99
8	EnN	0.5‒50	*y*=11125.4*x*+5638.8	0.99973	0.8	2.5	44.58
9	ImN+IdN	0.5‒50	*y*=31401.2*x*+514.4	0.99861	0.8	2.5	41.50
10	LaN	1.0‒50	*y*=10096.9*x*+1671.2	0.99833	1.5	5.0	38.91
11	Eu	1.0‒50	*y*=33206.8*x*+104.9	0.99894	1.5	5.0	38.84
12	HnN	0.5‒50	*y*=8551.7*x*‒3097.7	0.99919	0.8	2.5	33.77
13	St	2.0‒50	*y*=20318.3*x*‒1751.8	0.99977	3.0	10.0	30.26
14	Sp	5.0‒50	*y*=8454.2*x*‒640.7	0.99780	4.0	15.0	28.95
15	Ir	5.0‒50	*y*=2569.1*x*‒1780.5	0.99862	8.0	25.0	24.19
16	Sv	5.0‒50	*y*=4156.5*x*‒2436.8	0.99777	8.0	25.0	22.57
17	Sc	5.0‒50	*y*=9133.6*x*‒977.0	0.99996	8.0	25.0	23.30
18	EuN	0.5‒50	*y*=49642.3*x*+2209.9	0.99969	0.8	2.5	47.76
19	StN	5.0‒50	*y*=9330.5*x*+4833.7	0.99989	8.0	25.0	34.17
20	SpN	5.0‒50	*y*=5981.4*x*‒4522.1	0.99945	8.0	25.0	34.47
21	Us	5.0‒50	*y*=2956.7*x*‒738.5	0.99915	8.0	25.0	35.14
22	Re	5.0‒50	*y*=3240.6*x*+385.7	0.99524	8.0	25.0	30.51
23	SvN	5.0‒50	*y*=6866.6*x*+290.6	0.99942	4.0	15.0	23.82
24	IrN	5.0‒50	*y*=8510.5*x*‒2032.7	0.99867	8.0	25.0	26.62
25	ScN	5.0‒50	*y*=2823.5*x*‒2151.6	0.99836	8.0	25.0	22.41
26	Sk	2.0‒50	*y*=5586.3*x*+490.3	0.99630	3.0	10.0	16.32
27	ReN	5.0‒50	*y*=3956.3*x*+201.1	0.99876	8.0	25.0	40.54
28	UsN	5.0‒50	*y*=4269.1*x*‒1747.5	0.99976	8.0	25.0	35.10
29	Hs	0.5‒50	*y*=46828.3*x*+1671.4	0.99832	0.8	2.5	19.46
30	Em	0.2‒50	*y*=78071.9*x*+2183.4	0.99867	0.3	1.0	21.84
31	Lc	0.1‒50	*y*=194483.0*x*+4496.9	0.99933	0.2	0.5	28.93
32	EmN	0.5‒50	*y*=45288.1*x*+1928.7	0.99984	0.8	2.5	21.80
33	HsN	1.0‒50	*y*=11093.7*x*+257.6	0.99904	1.5	5.0	30.05
34	LcN	1.0‒‒50	*y*=9142.0*x*‒0.5	0.99976	1.5	5.0	33.96

*y*： peak area； *x*： mass concentration， ng/mL. The unit μg/kg is converted according to sample pretreatment process.

### 2.3 方法学验证

#### 2.3.1 线性范围、检出限和定量限

以基质匹配标准溶液中各PAs的质量浓度为横坐标*x*（ng/mL），以定量离子的峰面积为纵坐标*y*，绘制基质匹配标准曲线。如表3所示，35种PAs在各自范围内线性关系良好，根据干茶空白样品的3倍信噪比确定方法的检出限（LOD）、10倍信噪比确定方法的定量限（LOQ），LOD和LOQ分别为0.2~8.0 µg/kg和0.5~25.0 µg/kg（见表3）。

#### 2.3.2 回收率与精密度

以空白茶叶为基质分别进行1、2和5倍定量限水平的加标试验，进行准确度与精密度验证，每个加标水平重复3次。结果如表4所示，分别有91%、89%和89%的化合物平均回收率为70%~130%，且平行实验RSD小于20%（Em和Lc的RSD小于30%）。表明所建立的分析方法结果可靠，符合《食品安全国家标准 化学分析方法验证通则》（GB 5009.295-2023），可用于实际样品中35种PAs的定量分析。

**表4:** 干茶基质中35种PAs的加标回收率和相对标准偏差

Compound	1 fold-LOQ		2 fold-LOQ		5 fold-LOQ	
	Recovery/%	RSD/%	Recovery/%	RSD/%	Recovery/%	RSD/%
Im	77.29	8.40	74.00	7.81	70.77	1.65
Rn	73.23	2.83	75.02	3.92	71.02	2.36
Id	72.51	5.54	71.32	2.49	71.89	3.02
La	75.46	6.20	75.02	10.46	71.50	1.93
En	76.56	0.80	75.43	7.01	74.47	4.25
Hn	52.56	6.47	55.50	11.20	64.36	3.23
RnN	89.72	2.12	86.54	1.21	87.82	2.28
EnN	86.17	7.96	85.35	7.23	84.84	0.11
ImN+IdN	90.12	2.50	90.33	4.90	88.46	0.87
LaN	86.33	2.41	83.22	8.93	83.48	1.28
Eu	68.52	8.01	66.92	9.46	68.47	3.56
HnN	78.40	3.57	78.92	11.88	79.20	1.91
St	68.88	7.33	60.10	14.92	59.84	7.45
Sp	104.83	9.73	74.43	15.79	70.78	0.67
Ir	80.66	19.14	70.75	5.86	75.83	6.86
Sv	79.40	11.35	73.07	17.40	70.84	1.77
Sc	70.03	7.16	60.82	7.76	65.36	2.85
EuN	90.46	4.06	93.64	3.38	94.13	8.77
StN	76.01	17.92	78.13	16.45	78.81	4.46
SpN	78.81	19.81	76.06	12.69	81.29	5.01
Us	71.69	8.81	70.29	7.97	70.49	6.87
Re	75.85	17.86	78.37	1.75	71.51	4.47
SvN	85.33	10.10	83.44	5.51	82.32	0.48
IrN	90.31	9.42	84.74	5.89	85.63	9.30
ScN	85.41	8.42	104.88	13.53	91.32	4.85
Sk	119.47	8.82	111.97	8.43	109.40	1.55
ReN	94.96	12.04	96.95	6.23	101.59	7.41
UsN	96.71	8.71	107.00	4.05	87.87	7.73
Hs	80.79	8.30	76.59	7.85	83.69	6.06
Em	82.45	22.02	75.10	20.89	73.43	3.33
Lc	96.43	27.55	90.90	10.30	100.33	5.50
EmN	111.99	6.55	98.32	16.82	98.21	2.58
HsN	70.38	12.56	82.18	7.95	90.26	1.14
LcN	76.63	4.47	84.55	5.65	85.45	3.76

### 2.4 干茶样本中35种PAs的水平

应用本研究开发的分析方法分析了2023年在我国云南省和福建省当地茶园采摘的51份干茶样本，其中包括21份黑茶样本和30份红茶样本。在黑茶样本中有4份检出En，无其他PAs检出，样本检出率为19.05%，红茶样本中无PAs检出。黑茶样本中PAs的检出率高于红茶样本，与先前我国茶叶中PAs存在水平的研究结果一致^［30］^。这可能是由于大多数黑茶由机器采摘的鲜叶加工而成，采茶过程中难以避免地混入了含有PAs的杂草。所检出的4份黑茶中En最高含量为15.48 μg/kg，其次为8.67、8.01和5.07 μg/kg。所有样本中PAs含量均未超过欧盟限值标准对干茶样本中35种PAs总含量的最大限量（150 μg/kg）。

## 3 结论

本研究基于UHPLC-MS/MS建立了干茶样本中35种PAs的定量分析方法。33种PAs实现色谱分离和单独定量，可以更加准确地确定PAs的单独含量而不只是加和定量。应用该方法分析了云南省和福建省的21份黑茶和30份红茶样本中PAs存在水平，有4份样本检出。本研究的PAs定量分析方法可为茶叶中PAs的组成、含量及风险评估提供技术支撑。
